# Pre-operative immunotherapy with tumor cryoablation (cryo) plus ipilimumab (ipi) induces potentially favorable systemic and intratumoral immune effects in early stage breast cancer (ESBC) patients

**DOI:** 10.1186/2051-1426-3-S1-O6

**Published:** 2015-08-14

**Authors:** David B Page, Adi Diab, Jianda Yuan, Zhiwan Dong, Stephen B Soloman, Sujata Patil, Clifford A Hudis, Jedd D Wolchok, Larry Norton, Heather L McArthur

**Affiliations:** 1 Breast Medicine Service, Memorial Sloan Kettering Cancer Center, New York, New York, USA; 2 Department of Melanoma Medical Oncology, MD Anderson Cancer Center, Houston, Texas, USA

## Background

In mice, cryo plus checkpoint blockade facilitates tumor antigen release, T-cell priming, and improved survival [1]. Here, we assess immune response in ESBC patients using biomarkers that have been attributed to clinical benefit following checkpoint blockade [2-5].

## Methods

Women with ESBC were treated 7-10 days preceding mastectomy with either cryo (n=6), single-dose ipi at 10mg/kg (n=6), or cryo+ipi (n=6) [6]. From serial blood (baseline & 1-month post-mastectomy) and tumor (biopsy & mastectomy), fold-changes following cryo+ipi versus monotherapy were compared (Wilcoxon rank-sum) across the following measures: Ki67+ or ICOShi T-cells [2] and intratumoral T-effector/T-regulatory [3] cells by flow cytometry, plasma Th1/Th2 cytokines [4] (Meso Scale Discovery), and intratumoral T-cell expansion by immunohistochemistry [5] and T-cell receptor (TCR) deep sequencing (ImmunoSEQ) [5].

## Results

Cryo+ipi generated greater increases in peripheral Ki67+CD4+ (p=0.05), Ki67+CD8+ (p=0.05), ICOShiCD4+ (p=0.005), and ICOShiCD8+ (p=0.005) cells. The intratumoral T-effector/regulatory ratio was higher following cryo+ipi, but only when Ki67-gated (p=.01). Cryo+ipi generated greater increases in IL-2 (p=.01), IFNγ (p=.06), and IL-5 (p=.09). Despite negligible intratumoral changes by immunohistochemistry, cryo+ipi generated more high-magnitude (~1000 amplicon) clonal expansions by TCR sequencing (medians: 52 v. 3 clones).

## Conclusions

Cryo+ipi is associated with potentially favorable immunologic effects. Ki67-gating and TCR sequencing may identify intratumoral changes otherwise undetectable by flow or IHC.
